# The state of neuro-oncology during the COVID-19 pandemic: a worldwide assessment

**DOI:** 10.1093/noajnl/vdab035

**Published:** 2021-02-20

**Authors:** Maciej M Mrugala, Quinn T Ostrom, Shelley M Pressley, Jennie W Taylor, Alissa A Thomas, Jeffrey S Wefel, Scott L Coven, Alvina A Acquaye, Chas Haynes, Sameer Agnihotri, Michael Lim, Katherine B Peters, Erik P Sulman, Joanne T Salcido, Nicholas A Butowski, Shawn Hervey-Jumper, Alireza Mansouri, Kathy R Oliver, Alyx B Porter, Farshad Nassiri, David Schiff, Erin M Dunbar, Monika E Hegi, Terri S Armstrong, Martin J van den Bent, Susan M Chang, Gelareh Zadeh, Milan G Chheda

**Affiliations:** 1 Department of Neurology, Mayo Clinic, Scottsdale, Arizona, USA; 2 Department of Medicine, Epidemiology & Population Sciences, Baylor College of Medicine, Houston, Texas, USA; 3 Society for Neuro-oncology, Houston, Texas, USA; 4 Department of Neurological Surgery, University of California, San Francisco, California, USA; 5 Department of Neurological Sciences, University of Vermont Larner College of Medicine, Burlington, Vermont, USA; 6 Departments of Neuro-Oncology and Radiation Oncology, University of Texas MD Anderson Cancer Center, Houston, Texas, USA; 7 Division of Pediatric Hematology/Oncology, Indiana University School of Medicine, Indianapolis, Indiana, USA; 8 Neuro-oncology Branch, Center for Cancer Research, National Cancer Institute, National Institutes of Health, Bethesda, Maryland, USA; 9 Department of Neurological Surgery, University of Pittsburgh, Pittsburgh, Pennsylvania, USA; 10 Department of Neurosurgery, Johns Hopkins University, Baltimore, Maryland, USA; 11 Departments of Neurology and Neurosurgery, Duke University, Durham, North Carolina, USA; 12 Department of Radiation Oncology, NYU Grossman School of Medicine, New York, New York, USA; 13 Brain and Spine Tumor Center, Laura and Isaac Perlmutter Cancer Center, NYU Langone Health, New York, New York, USA; 14 Pediatric Brain Tumor Foundation, Asheville, North Carolina, USA; 15 Department of Neurosurgery, Penn State Health, Hershey, Pennsylvania, USA; 16 International Brain Tumour Alliance, Tadworth, UK; 17 Departments of Neurologic Surgery and Hematology Oncology, Mayo Clinic, Phoenix, Arizona, USA; 18 Division of Neurosurgery, Department of Surgery, University of Toronto, Toronto, Ontario, Canada; 19 Departments of Neurology, Neurological Surgery and Medicine, University of Virginia School of Medicine, Charlottesville, Virginia, USA; 20 Piedmont Brain Tumor Center, Atlanta, Georgia, USA; 21 Neuroscience Research Center, Lausanne University Hospital and University of Lausanne, Epalinges, Switzerland; 22 Brain Tumor Institute, ErasmusMC Cancer Institute, Rotterdam, The Netherlands; 23 Departments of Medicine and Neurology, Washington University School of Medicine, St. Louis, Missouri, USA

**Keywords:** clinical trial enrollment, COVID-19, neuro-oncology outcomes

## Abstract

**Background:**

It remains unknown how the COVID-19 pandemic has changed neuro-oncology clinical practice, training, and research efforts.

**Methods:**

We performed an international survey of practitioners, scientists, and trainees from 21 neuro-oncology organizations across 6 continents, April 24–May 17, 2020. We assessed clinical practice and research environments, institutional preparedness and support, and perceived impact on patients.

**Results:**

Of 582 respondents, 258 (45%) were US-based and 314 (55%) international. Ninety-four percent of participants reported changes in their clinical practice. Ninety-five percent of respondents converted at least some practice to telemedicine. Ten percent of practitioners felt the need to see patients in person, specifically because of billing concerns and pressure from their institutions. Sixty-seven percent of practitioners suspended enrollment for at least one clinical trial, including 62% suspending phase III trial enrollments. More than 50% believed neuro-oncology patients were at increased risk for COVID-19. Seventy-one percent of clinicians feared for their own personal safety or that of their families, specifically because of their clinical duties; 20% had inadequate personal protective equipment. While 69% reported increased stress, 44% received no psychosocial support from their institutions. Thirty-seven percent had salary reductions and 63% of researchers temporarily closed their laboratories. However, the pandemic created positive changes in perceived patient satisfaction, communication quality, and technology use to deliver care and mediate interactions with other practitioners.

**Conclusions:**

The pandemic has changed treatment schedules and limited investigational treatment options. Institutional lack of support created clinician and researcher anxiety. Communication with patients was satisfactory. We make recommendations to guide clinical and scientific infrastructure moving forward and address the personal challenges of providers and researchers.

**Key Points**

- Clinical trial suspension, including phase III trials, was a hallmark of the pandemic.
- Practitioners and researchers perceive significant personal risk in doing their jobs.
- No consensus exists about risks for SARS-CoV-2 infection in neuro-oncology patients.

**Importance of the Study**

This is the first international study of the impact of the COVID-19 pandemic on the field of neuro-oncology. We observed changes in treatment options for patients with brain and spine tumors, as well as burdens on clinicians and researchers. We highlight major challenges in the field, including suspension of clinical trials, financial pressures for practitioners to see patients in person, and unmet safety concerns and high anxiety of practitioners and scientists. We also identified positive outcomes in perceived quality of communication with colleagues, patients and families, and reduced travel and expenses for patients. This work serves as a benchmark assessment of the field during the early days of the pandemic.

The COVID-19 pandemic has created many challenges for healthcare.^1^ Neuro-oncology, which focuses on treating patients with primary and metastatic brain and central nervous system (CNS) tumors and neurologic complications of cancer, has faced challenges, particularly in maintaining quality patient care and conducting clinical trials and laboratory research.

The provider pool in neuro-oncology is relatively small. In 2018, there were 2600 members in the Society for Neuro-Oncology (SNO) database, of which 1040 (40%) were clinical members, including physicians, nurses, and nurse practitioners. Equally small numbers of neuro-oncologists practice in Europe, Asia, and the rest of the developed world. In the United States, approximately 25 000 malignant primary brain tumors are diagnosed annually.^2^ There are significant differences in incidence by world region, with the highest incidence of malignant brain tumors in Northern Europe and Canada and the lowest in Southeast Asia.

Gliomas are the most aggressive primary brain tumor, and they make up the majority of diagnoses in neuro-oncology. Standard treatment for high-grade gliomas includes surgical resection, followed by radiation and temozolomide, an alkylating chemotherapy, and more recently, adjuvant use of tumor-treating fields. Often during the course of care, patients receive dexamethasone, which reduces edema; bevacizumab, a monoclonal antibody that reduces angiogenesis and edema; and/or lomustine, an alkylating chemotherapy. Radiation, temozolomide, and lomustine all commonly cause myelosuppression and lymphopenia.^3,4^ Immunotherapy is not approved for primary brain tumors; it remains under investigation and is sometimes used as an off-label treatment at the time of tumor recurrence.

The pandemic has required re-organization of clinic visits, treatment and diagnostic testing schedules, and the development of new processes managing therapy-related complications.^5^ Patient management guidelines^6–8^ have emerged, and discussions have centered on how to approach brain tumor patient treatment in resource-limited settings and when significant exposure risk to patients and health care providers exists.^9^ These recommendations notwithstanding, it remains unknown as to what practically changed during the pandemic. The pandemic has also affected medical teams. While physicians and allied professionals are accustomed to dealing with stress and dying patients, the burden of these new challenges has been unprecedented on professional and personal lives. Clinicians need support during the COVID-19 pandemic,^10^ and guidelines exist for managing psychological stress and maintaining healthy physical and mental states.^11^ However, we lack basic measurements of what clinicians and researchers are experiencing.

Medical education was restructured so medical students, residents, and fellows may continue learning to take care of patients and advance through their training programs.^12,13^ Many programs are facing financial challenges that jeopardize the continuity of their training mission and the ability to accept new trainees.

Finally, scientists in neuro-oncology, attempting to understand the mechanisms underlying brain tumor development or identify new treatments, have had to stop or postpone costly experiments and are now at risk of losing funding to continue supporting these efforts

Here, we sought to address these issues and evaluate the perceived effects of these changes on neuro-oncology patient care. We asked the international community of healthcare providers, scientists, and trainees in the field to share their experiences during the pandemic. This survey tool is not a method to identify the root causes of the problems we identify. This framework will guide further studies and recommendations on how to best take care of CNS cancer patients, support clinical caregivers, and identify opportunities for institutions to continue to advance their research mission. These findings and principles will be broadly applicable to other oncology specialties and those caring for patients with chronic disease.

## Methods

The survey (Supplementary File 1) was developed by the SNO COVID-19 task force, composed of adult and pediatric oncologists, surgeons, radiation oncologists, laboratory scientists, and patient advocates. The task force generated a set of general questions for all the participants and in addition, we created several pathways based on self-designation (eg, clinician [neuro-oncologist, neurosurgeon, or radiation oncologist], scientist, trainee [graduate students, postdocs, residents, and fellows], social worker, training program director). Participants who were members of more than one group were asked to complete all applicable pathways. Topics were selected based on the interests of the committee and included questions about personal physical/mental health, institutional response, clinical practice changes, and individual career outcomes. Questions were a combination of binary response, multiple choice, Likert scale, and free response. For some topics, respondents were asked an overall question and then provided follow-up binary questions for more detailed responses. The time period for which questions were asked was since the beginning of the COVID-19 pandemic in their respective countries. The survey was available in English only. We sent email survey invitations to members of 21 neuro-oncology organizations (Supplementary File 2) on 6 continents and advertised the survey on multiple social media platforms. The survey was administered through Survey Monkey (www.surveymonkey.com) from April 24 to May 17, 2020 and contained 134 questions, presented in a modular format with smart logic. We used R 4.0.0 software to generate summary statistics and perform statistical analyses. Categorical variables were compared using chi-square tests, and continuous variables were compared using *t*-tests (when only 2 comparison groups) or ANOVA (when more than 2 comparison groups). Data on sex, age, and ethnicity of the respondents were not collected for this survey.

## Results

**Figure 1.**
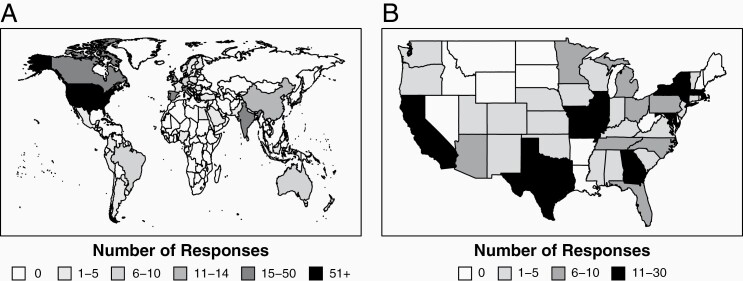
Number of respondents by (A) country and (B) US state.

A total of 582 responses were received. Respondents were located in the United States (258, 45%) and internationally (324, 55%; Figure 1). Of international respondents, Europe (124/582, 21.3%) and Asia (91/582, 15.6%) were the most represented regions, and within these, Spain (37/582, 6.4%) and Japan (19/582, 3.3%) were the most common countries of residence (Figure 1). Respondents representing those with direct patient contact included 53.4% physicians (311/582), 2.6% nurse practitioners and physician assistants (15/582), 0.7% nurses (2/582), and 0.2% social workers (1/582); and 7.9% were researchers (46/582; Table 1). Of the clinicians, 71.0% treated adults (341/582), 11.3% treated children (54/582), and 17.7% treated both (85/582). Within the United States, most clinical practitioners were located at academic centers (79.0% of non-researchers or 452), compared to private practice (15.2% of non-researchers or 70).

**Table 1.** Characteristics of Survey Respondents

Questions	*N* = 582
Do you primarily consider yourself a*: (%)	
* *Clinician	227 (39.0%)
* *Clinician scientist	174 (29.9%)
* *Scientist	39 (6.7%)
* *None of the above	5 (0.9%)
* *No response	137 (23.5%)
Occupation (%)	
* *Physician	311 (53.4%)
* Neuro-Oncologist (includes medical oncology, neurology, or pediatrics)*	169 (29.0%)
* Neurosurgeon*	94 (16.2%)
* Radiation Oncologist*	48 (8.2%)
* *Scientist or researcher	46 (7.9%)
* *Advanced practice practitioner (nurse practitioner, physician assistant)	15 (2.6%)
* *Trainee (includes graduate students, postdocs, residents, and fellows)	11 (1.9%)
* *Nurse	4 (0.7%)
* *Occupational, speech, or physical therapist	2 (0.3%)
* *Social worker	1 (0.2%)
* *Other	13 (2.2%)
* *No response	179 (30.8%)
Do you primarily treat adults or children? (%)	
* *Adult patients	341 (58.6%)
* *Both adult and pediatric patients	85 (14.6%)
* *I do not provide direct care to patients	81 (13.9%)
* *Pediatric patients	54 (9.3%)
* *Neither	11 (1.9%)
* *No response	10 (1.7%)
Where do you primarily practice? (%)	
* *Academic center (main campus or its satellite locations)	452 (77.7%)
* *Private practice	70 (12.0%)
* *Other	40 (6.9%)
* *No response	20 (3.4%)
World region (%)	
* *United States	258 (44.3%)
* *Europe	124 (21.3%)
* *Asia	91 (15.6%)
* *Other	109 (18.7%)

*This question was required to move forward with the rest of the survey (Supplementary File 1). If this was not answered, the respondent did not move past this question.

### Neuro-oncology Relevant Risk Factors for COVID-19

Regarding beliefs of COVID-19 risk, 50.3% of respondents believed that neuro-oncology patients, before any treatment, are at increased risk for contracting the virus (180/487); 48.9% believed that steroids increase susceptibility to SARS-CoV-2 infection (173/487), but 43.8% reported they were unsure and needed more evidence (155/487). Twenty-eight percent believed that radiotherapy increases infection risk (100/487). Forty-six percent believed that temozolomide use increases susceptibility (165/487), and 21.6% believed that immunotherapy increases the risk (77/487).

### Effects on Clinical Practice and Patients

The pandemic required changes to clinical practice and clinical trial opportunities, and in some cases, clinicians reported pressures related to billing. While practitioners voiced concerns about the emotional well-being of their patients, there were perceived positive benefits to patients in terms of communication of treatment plans and other aspects of care.

**Figure 2.**
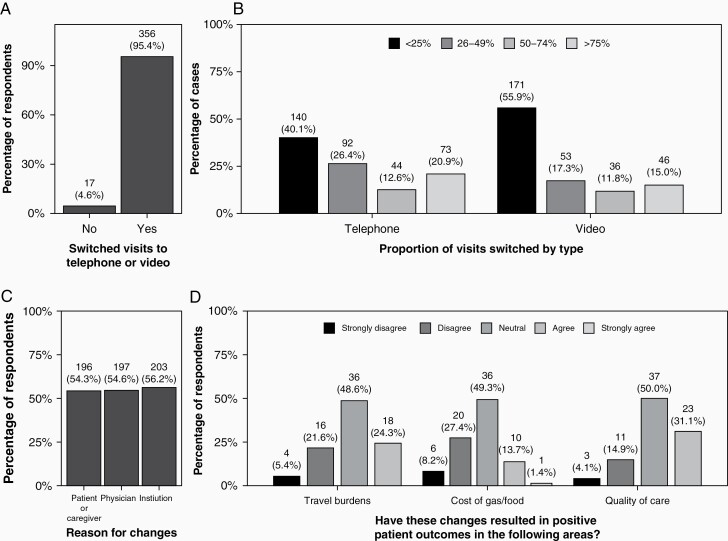
(A) Proportion of respondents reporting increased use of telephone and video visits; (B) percentage of cases transferred to video or telephone; (C) primary person requesting change (respondents may have selected multiple options); and (D) proportion reporting positive patient outcomes as a result of these changes.

Ninety-four percent of participants reported changes in clinical practice due to the pandemic (361/386). The proportion reporting changes was highest in the United States (96%, 169/177), compared to Europe (95%, 87/92) and Asia (82%, 48/59, *P* = .003). Regarding survival, 44.5% thought practice changes due to the pandemic would reduce survival, outside of any direct effect of SARS-CoV-2 infection in their patients (151/339). This perception was highest in Asia (54.0%, 30/53) compared to Europe (45.5%, 35/77) and the United States (39.8%, 64/131), but not statistically significant (*P* = .101). Almost all respondents transitioned to the use of video or telephone visits for some aspects of clinical care (95.4%, 356/373; Figure 2). Use of telemedicine was slightly lower in Asia (85.2%, 48/56), compared to Europe (97.7%, 86/88) and the United States (98.3%, 171/174, *P* = .001). A majority (56.8%, 192/338) reported this transition was at least somewhat difficult; however, 80.1% received adequate information technology support (285/351).

While practitioners greatly used telemedicine, 85.9% (322/375) canceled what they deemed nonessential patient visits, and 16.2% moved patient visits at least 2 months into the future (61/376). Of these, 55% of scheduling changes were requested by patients or caregivers (206/371). Regarding chemotherapy scheduling, 12.3% (35/285) of practitioners moved inpatient chemotherapy by up to 2 weeks and another 6.7% moved it out by up to a month (19/285). Regarding referrals, 27.2% (101/372) practitioners referred patients to other institutions as a result of pandemic pressures, and 65.4% (233/356) changed MRI schedules for their patients. Referrals to other providers were most frequently reported in Asia (43.9, 25/57%), as compared to Europe (17.0%, 15/88) or the United States (24.4%, 42/172, *P* = .002).

Practitioners remained concerned about emotional and palliative care of patients. Eighty percent of clinical respondents noticed increased anxiety and depression in their patients. Almost 20% of respondents noted an increasing need for palliative care consults. Approximately 35% reported increased frequency in the discussion of end-of-life issues since the beginning of the pandemic, but only 6.8% of end-of-life discussions were initiated by patients.

When asked whether there may be any positive aspects of the pandemic, 88% agreed or strongly agreed (415/471) that new technologies applied toward patient care were a positive outcome. In fact, 84% (403/474) agreed or strongly agreed that virtual meetings, including tumor boards, were very helpful. Remarkably, 74.3% (347/ 467) of respondents agreed or strongly agreed there was increased satisfaction of patients and families, due to decreased burdens of spending time and money traveling to appointments. This was highest in Asia (75%, 54/72), compared to Europe (67%, 69/103) and the United States (67.9%, 167/226, *P* = .0152). About 59.5% (275/462) of practitioners also agreed or strongly agreed that the quality of care exchange would be positively affected by the change in norms during the pandemic. This was highest in Asia (63.4%, 45/71), compared to the United States (62.2%) or Europe (50.0%, 50/100, *P* = .0044).

In terms of reimbursement, 25.9% (83/321) of respondents stated they were not billing for technology-assisted visits. This was highest in Asia (56.8%, 25/44) and Europe (47.3%, 36/76), compared to the United States (6.9%, 11/160, *P* < .001). Overall, 29.4% (94/320) of respondents had not received effective support in billing for these types of visits. Among those who reported billing for telephone and video visits, 82.8% (193/233) reported receiving effective support as compared to only 35.5% (27/76) of those who did not bill for telephone and video visits (*P* < .001). Additionally, nearly 10% (28/322) of respondents felt pressured by their institution to do in-person visits, because of billing needs and not because patient care necessitated an in-person visit; this pressure was greatest in Asia (15.9%, 7/44), followed by the United States (7.9%, 11/156) and then Europe (5.3%, 4/76, *P* = .104).

**Figure 3.**
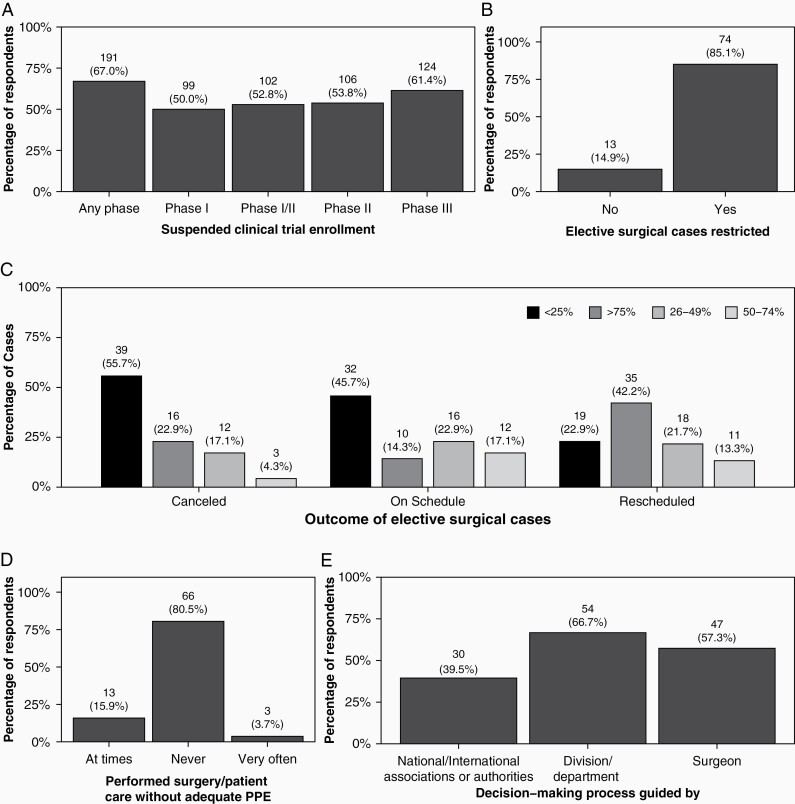
(A) Proportion of respondents reporting suspending clinical trials overall and by phase; (B) proportion of respondents reporting restrictions on performing elective surgeries; (C) outcome of elective surgical cases; (D) proportion reporting performing clinical care without appropriate PPE; and (E) major factors guiding decision making for elective surgeries.

Notably, 67% of practitioners had suspended enrollment for any clinical trial (191/285), with 50% for phase I trials (99/198), 52% for phase I/II trials (102/193), 53% for phase II trials (106/197), and 62% for phase III trials (124/202; Figure 3). The effect of COVID-19 on non-therapeutic trials was not measured by this survey, and it is not possible to assess the effect on other human subjects research. Regarding changes to treatment, 27.4% (46/168) of practitioners altered standard of care regimens, and approximately 20% changed the timing or dosing of infusions of bevacizumab (32/162). Forty-three percent (71/165) noted they were being more careful using myelotoxic regimens because of unknowns about SARS-CoV-2 infection and those at risk. Nine percent (14/156) of practitioners stopped off-label regimens and 23.1% (36/156) reduced their frequency. Pediatric oncologists had not changed their practice in any significant way.

**Figure 4.**
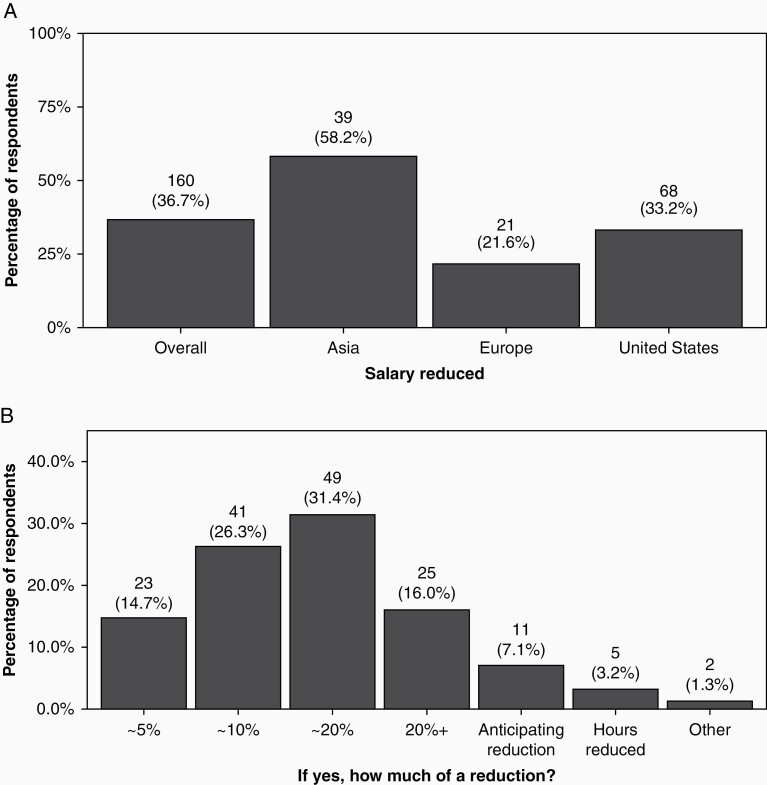
(A) Overall proportion of respondents reporting salary reduction and proportion reporting reduction by region and (B) amount of salary reduction.

Twenty-eight percent of respondents believed that radiotherapy increases susceptibility to SARS-CoV-2 (100/363). Radiation oncology plans for high-grade glioma were unchanged in 77.1% (37/48); 16.7% of cases were delayed by 2–4 weeks (8/48). For other indications, less than 40% of radiation oncology plans remained unchanged (18/48); approximately 58.3% (28/48) were delayed by at least 2 weeks. In cases of modified radiation plans, 68% (17/25) were changed to shorter courses with higher daily doses or shorter courses with a lower total dose (20%, 2/25; Figure 4).

In terms of changes in surgical practice, an average of 60% of elective cases were rescheduled into the future, and remarkably, an average of 37% of elective procedures were canceled. About 14.3% (12/84) of cases that were planned with an endonasal approach were converted to craniotomy because of guidelines from respective institutions and surgical organizations. Notably, in cases where there was inadequate personal protective equipment (PPE), approximately 19.5% (16/82) of respondents reported already scheduled surgical procedures proceeding at times or very often.

### Effects on Laboratory Research

Neuro-oncology laboratory-based research significantly slowed during the early months of the pandemic. Overall, 63% of respondents closed their laboratories (34/54), with the number being 78.6% in the United States (22/28); 72.7% of respondents stopped long-term experiments (32/44). Respondents were not asked how long these laboratories were closed. Regarding funding prospects, 47.1% (24/51) were “very concerned” about their own research funding because of the economic strains associated with the pandemic. For 34.9% (22/63), grant submission deadlines had been postponed, while 48.7% (19/39) believed that pandemic-related changes gave them more time to write scientific manuscripts. Nearly 30% of respondents (17/57) reported their academic careers would be altered. For the US-based respondents, only 13.3% (2/15) reported that a visa status was at risk because of pandemic-related pressures or policies; of note, the survey was conducted prior to the June 22, 2020 presidential executive order suspending H1B and other foreign worker visas.

### Work Hours, Salary, Benefits, and Job Security

**Figure 5.**
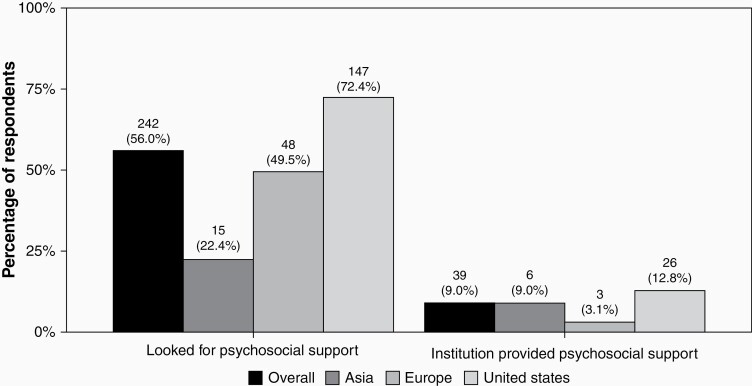
Proportion of respondents that looked for psychosocial support and who were offered support by their institution, overall and by world region.

While approximately 30% (125/433) of respondents reported increased work hours since the pandemic started, 37% (160/436) had their salary temporarily reduced. This was higher for respondents who primarily treated adults (38%, 107/279) as compared to children (23%, 10/43, *P* = .0016). Of those with salary reductions, 53.6% (74/138) had salaries reduced by at least 20%. Four percent (16/434) of respondents had been furloughed or fired. The majority of respondents in Asia (58.2%, 39/67) reported salary reduction, as compared to 33.2% US respondents (68/205) and 21.6% European respondents (21/97, *P* < .001; Figure 5). Thirty-seven percent of clinical practitioners (85/228) and 57% of researchers (4/7) reported a severe or moderate to severe fear of loss of job security (*P* = .4291). This was higher in private practice (51.1%, 24/47) as compared to academic institutions (37%, 87/238) and was highest in the United States (46.4%, 65/140), compared to Europe (34.5%, 20/58) or Asia (37%, 20/55, *P* = .1303).

### Well-being

The majority of respondents (75.7%, 206/272) reported increased stress during the early months of the pandemic. This was higher in the United States (82%, 107/130), compared to Asia (61.2%, 34/52) and Europe (58.8%, 34/52, *P* = .190). Regarding personal fears, 81.4% (188/231) of practitioners had moderate or severe fear for the health of their own families, specifically because of their clinical duties; 71.4% (5/7) of researchers reported that they were fearful for their health, specifically because of their research duties. Of all respondents, 53.8% (161/299) had a moderate or severe concern about transmitting SARS-CoV-2 to their family, and 37.4% (111/297) had moderate or severe concerns about transmitting to other health care workers. “Severe concern” about one’s own health and survival was reported in approximately 10% (27/298) of all respondents. While a majority faced increased stress, only 56% (242/432) had psychosocial support offered by their institution. Institutional psychosocial support availability varied significantly by region, with 72% of US respondents (147/203) reporting they were offered support, compared to 49.5% in Europe (48/97) and 22.4% in Asia (15/67, *P* < .001).

### Training

Approximately half of fellowship program directors worried about funding for their fellows because of the pandemic (20/38), and 40% (14/35) reported concerns they would not be able to completely fill neuro-oncology fellowship slots for 2021.

## Discussion and Recommendations

There have been several consensus statements and commentaries regarding neuro-oncology care during the pandemic,^6,7,14^ and analyses of the caregiver, not-for-profit and brain tumor charity experiences.^15,16^ Our international survey provides data that reveal the impact of the pandemic on the practice of neuro-oncology care, its effects on clinical caregivers and patients, and research (Table 2). First, the clinical practice dramatically changed in the first several months of the pandemic. Transition to telemedicine occurred almost universally, although it varied by region. The closure of clinical trials, including phase III, was remarkable, especially given the poor standard of care options for patients with malignant gliomas. These closures may reflect a lack of clinical research administrative support due to financial or safety concerns, a lack of flexibility in government and pharmaceutical trial contracts, a general lack of support by institutions themselves, the inability of institutions to safely promote social distancing, difficulty obtaining operating room specimens, or other causes. We also wonder whether this reflects a feeling among providers that what we have to offer patients in terms of treatment does not carry a significant chance of benefit and therefore does not meet a threshold of risk to participate. Comparing these findings to other cancers and diseases will be helpful to identify the root cause of this problem. A study of Canadian oncologists early in the pandemic similarly found that half of the respondents reported a cessation of clinical trial accrual.^17^ Public pressure by patient advocacy groups, as well as more institutional experience with how to manage clinical trial complexities during the crisis, may enable trials to remain open in the future. It was remarkable that clinical practitioners believed that patient satisfaction has increased because of improved communication and travel reduction. This improved satisfaction may also be due to reduced delays in scheduling, which we did not assess. There may be lessons here on how to improve care beyond the pandemic and it will be important to analyze patient and caregiver assessments to corroborate this perception of the care team. For example, respondents deemed virtual platforms beneficial for tumor boards and communication with colleagues and this may represent a lasting positive outcome of this pandemic. In addition, such patient and caregiver-facing studies should evaluate whether the usefulness of technology-assisted visits is evident across the board, or if the elderly or patients in resource-poor settings who find it more difficult to get access to care benefit the most. Lastly, these results provide a platform for future outcomes research aimed at assessing whether subsets of virtual care are inferior, non-inferior, or superior than traditional health care delivery in terms of medical outcomes, patient/caregiver satisfaction, resource utilization, and financial ramifications to the health care system, families, and society. This may also be an occasion to revisit the general usefulness of procedures that were dropped during the pandemic.

**Table 2.** Key Findings and Recommendations for Institutions and COVID-19-Related Research Priorities for the Neuro-oncology Community

Key Findings
Clinical trial enrollment was impacted by the pandemic.
In some cases, telemedicine billing support for practitioners was inadequate.
Some practitioners felt pressure to do in-person visits.
Elective surgical practice changed.
Perception of increased anxiety in patients.
Respondents expressed concerns about their emotional well-being, safety for self and family, and financial impact from the pandemic.
Positive aspects of pandemic-based changes:
Technologies applied to patient care
Virtual meetings among colleagues
Perceived increase in patient satisfaction due to decreased time and money traveling to appointments
Major Recommendations
*Institutions*:
Modify and prioritize clinical trial infrastructure to ensure access for all patients.
Provide support for billing education for telephone and video visits.
Remove pressures on providers to see patients in-person when not clinically necessary.
Consider support for those with children and elder care responsibilities.
Provide means of psychological support to staff.
*Areas of further research*:
Effects of modified treatment schedules, in-person visit reductions, and surgical delays on patient outcomes.
COVID-19 risk factors and outcomes in neuro-oncology patient population as a function of treatment; laboratory studies in disease models.
Impact of financial changes on productivity and provider wellness.
Impact of financial changes on the conduct of basic science and clinical research.
Burdens as a function of provider and researcher gender and other demographics.

Next, the degree of practitioners’ worries for their own, and their families’ health because of potential exposures they encounter at work is remarkable. It was striking that 15% of surgical procedures proceeded despite inadequate PPE. Dogma is an early surgical intervention that is beneficial, particularly for patients with aggressive tumors. Many of these procedures were delayed. The impact on survival remains unknown.

A large percentage of respondents faced significant financial loss. Further work is required to determine the long-term effects of these financial changes on productivity and patient outcomes. We also note the significant pressures that exist for clinical practitioners and researchers alike that come from the nature of the pandemic itself. For example, institutions should take measures to support those with children and elder caregiving responsibilities. School closures or schools on altered schedules relying on home education are likely disproportionately more challenging for women. We did not assess this, but future studies should identify the gender differentials in terms of stress, productivity, and support received. Support may come from tenure clock delays, financial support for those with children, and technical help in the laboratory.

Additionally, the future of neuro-oncology research may be in question, as trainees in the clinic and the laboratory face challenges with funding and future training opportunities. While the economy as a whole is affected by the pandemic, we wonder how much might be lost in terms of advances in the field if trainees leave the pool of future neuro-oncology practitioners and researchers.

We recommend hospitals and insurance providers offer support for billing for video and telephone visits. Education on Medicare (in the United States) and other insurance reimbursement policies for telemedicine use should be improved. Ten percent of respondents felt pressured by their institution to continue to see patients in person because of billing considerations. While there may be more nuanced reasons for this that our survey did not capture, this compounds stress without improving patient care, and clinical societies should advocate toward the end of this practice. Beyond the perceived health risks of clinical practitioners and researchers, the degree of reported professional caregiver anxiety and depression should be addressed by institutions.

While there are early observations that cancer patients in general may be more vulnerable to developing COVID-19,^18–23^ this is not certain and there remain open questions for further research, specifically in the care of patients with brain tumors. There are strongly divergent opinions on whether or not having a brain tumor increases the risk of contracting the virus and whether or not temozolomide and standard steroid dosing affect susceptibility. Retrospective and laboratory-based studies may yield insights. Additionally, comorbidities in the brain tumor population, such as venous thrombosis and pulmonary embolism, or pulmonary disease in those with *Pneumocystis jirovecii,* may have ramifications for the susceptibility of this patient population to infection with SARS-CoV-2.

A study of this kind is not without limitations given the fluid nature of the pandemic and varied institutional, national, and international responses. Due to the way that the survey was advertised, it is not possible to calculate a response rate. The number of total responses represents a small proportion of membership in professional organizations that were contacted about participation, suggesting that only a small proportion of the neuro-oncology community was captured by this survey. The survey was offered only in English, which limited participation from non-English speakers. These factors introduce the possibility of selection bias. While we were able to capture the burden providers felt related to the angst over uncertainty and the well-being of their patients, staff, families, and themselves, we recognize that women and people of diverse backgrounds may have been impacted in ways we did not capture due to the lack of inclusion of these demographic variables. The xenophobia that emerged resulting in bias faced by Asian colleagues and patients reported elsewhere is unprecedented.^24^ Our study did not address aspects of discriminatory behavior as a result of the COVID-19 pandemic, nor were we able to assess the impact of stressors faced by women providers and single parents responsible for the care and education of their children.

Our work serves as a baseline appraisal of neuro-oncology during the early months of the pandemic. Evidence suggests practitioners are at risk for burnout. Clinical trial and off-label options are being reduced. Standard treatment options are being modified. New research efforts have been slowed. On the other hand, our assessment provides institutions and advocacy groups with a framework to intervene. As we all learn more, our hope is that such interventions will make oncologic care more efficient and improved on the other side of the pandemic.

## Funding

This work was supported by the National Institute of Neurological Disorders and Stroke of the National Institutes of Health (NIH) under award numbers R01 NS107833 and R01 NS117149 (to M.G.C.), grant 2015215 from the Doris Duke Charitable Foundation (M.G.C.), and the generosity of the friends and families of Larry Stark and Brett Pickle (M.G.C.). Q.T.O. is supported by a Research Training Grant from the Cancer Prevention and Research Institute of Texas (RP160097T). The funding sources had no role in any decisions regarding the survey questions, analysis, or writing of the manuscript.

## Supplementary Material

vdab035_suppl_Supplementary_Materials
