# Supplementary material for: The state of neuro-oncology during the COVID-19 pandemic: a worldwide assessment
Source: Neurooncol Adv. 2021 Feb 20;3(1):vdab035. doi: 10.1093/noajnl/vdab035 (PMC7928618; doi:10.1093/noajnl/vdab035)
Supplement: vdab035_suppl_Supplementary_Materials [file vdab035_suppl_supplementary_materials.pdf]

Participating organizations

The 21 participating neuro-oncology organizations included the Society for Neuro-Oncology (SNO); Asian Society for Neuro-Oncology (ASNO); European Association of Neuro-Oncology (EANO); Society for Neuro-Oncology Latin America (SNOLA); Society for Neuro-Oncology Sub-Saharan Africa (SNOSSA); Society of Austrian NeuroOncology (SANO); Belgian Association for Neuro-Oncology (BANO); Chinese Society of Neuro-Oncology; Egyptian Group of Neuro Oncology (EGNO); Association des Neuro-Oncologues d'Expression Francaise (ANOCEF); Neuro-Oncology Working Group of the German Cancer Society (NOA); Indian Society of Neuro-Oncology (ISNO); Italian Association of Neuro-Oncology (AINO); Japan Society for Neuro-Oncology (JSN); Korean Society for Neuro-Oncology (KSNO); Dutch Society for Neuro-Oncology (LWNO); Serbian Neurooncology Society; Grupo Español de Investigación en Neurooncología (GEINO); Swiss Group for Clinical Cancer Research, Working Group for Brain Tumours (SAKK); Noroonkoloji Dernegi (TNOD); and British Neuro-Oncology Society (BNOS). There were 344 individuals who indicated that they were members of SNO.

Society for NeuroOncology

NEURO-ONCOLOGY SURVEY COVID-19

1. Do you primarily treat:
    a. Pediatric Patients
    b. Adult Patients
    c. Both
    d. Neither
    e. I do not provide direct care to patients

2. Where do you primarily practice?
    a. Academic center (main campus or its satellite locations)
    b. Private practice-urban
    c. Private practice-rural
    d. Other (please specify)

3. I'm a member of the following neuro-oncology societies:
    a. ASNO
    b. EANO
    c. SNO
    d. SNOLA
    e. SNOSSA
    f. Other (please specify)

4. Do you practice in the United States of America?
    a. Yes
    b. No

5. In what state or U.S. territory do you currently work?

6. In what country do you practice?

7. Do you consider introduction or accelerated adoption of the following to be positive outcomes during/after the pandemic?
    a. New technologies brought to one's clinic/hospital, e.g., voice dictation, webcams, telehealth/video platforms (zoom, web-ex)
    b. Virtual meetings, e.g. tumor board, department meetings, etc.
    c. Virtual patient encounters (telehealth (video), phones)
    d. Billing/reimbursement platforms for virtual patient encounters
    e. Liberalized requirements for controlled substances (electronic prescribing, ability to prescribe 90-day or refills, etc.)
    f. Liberalized requirements for face-to-face encounters for refilling therapy referrals, electric wheelchairs, other Medicare/Medicaid services, etc.

Society for NeuroOncology

g. Patients & caregiver satisfaction secondary to lowered travel burdens, effort/cost for transport, effort/cost to caregiver for taking time off work to assist
h. Patient & caregiver satisfaction secondary to effort/cost for gas/food
i. Patient & caregiver satisfaction regarding the feasibility and the quality of the care exchange
j. Less family and visitors in clinic and the hospital

8. Do you consider any of the following to be negative outcomes during the COVID-19 pandemic?
    a. Patient safety
    b. Patient outcomes (e.g., tumor control, overall survival)
    c. Funding for my research
    d. Funding for my practice location to serve patients (patient services, keep clinics open, hospital staff)
    e. Less or slower advancement of my professional projects
    f. Less or slower advancement of the strategic initiatives of my department or institution
    g. Loss of health limiting your ability to practice medicine
    h. Loss of security in maintaining a job in medicine ("job security") and/or future promotion options
    i. Less or slower fund-raising (philanthropy) for me or my department or institution
    j. Less or slower of not-for-profit (charity) organizations ability to fundraise, and thus less monies available to patients
    k. Less or slower of pharma/industry grants for research, drug assistance programs, and related

9. Has your number of hours worked increased since the pandemic started?
    a. Yes
    b. No

10. Has your institution provided psychosocial support for you?
    a. Yes
    b. No
    c. If yes, (please specify)

11. Have you looked for psychosocial support during this time?
    a. Yes
    b. No
    c. Yes (please specify)

12. Do you feel that your stress has increased during this time?
    a. Yes
    b. No
    c. Yes, what has added to your stress (please specify)

13. Are you able to take care of basic needs (safety, family, transportation to institution, eating, sleeping, etc.?
    a. Yes

SNO Society for NeuroOncology

b. No
c. No, what has been ignored (please specify)

14. As a result of COVID-19 pandemic has your salary been temporarily reduced?
    a. Yes, by 5%
    b. Yes, by 10%
    c. Yes, by 20%
    d. No
    e. Yes, other (please specify)

15. As a result of COVID-19 pandemic has your institution implemented reductions in other benefits? Please check all that apply:
    a. Pension Plan
    b. 401K or 403B Contributions
    c. Health Insurance
    d. Vacation Fund
    e. Travel Fund
    f. Reimbursement account for dues, memberships, journal subscriptions
    g. No
    h. Other (please specify):

16. Have you been furloughed or fired as a result of the COVID-19 pandemic?
    a. Yes
    b. No

17. Are you at risk of being furloughed or fired as a result of the COVID-19 pandemic?
    a. Yes
    b. No
    c. I don’t know/not sure

18. Do you primarily consider yourself a:
    a. scientist
    b. clinician
    c. both clinician scientist
    d. none of the above

19. Are you fearful for you and/or your family’s health by your clinical duties in the face of the COVID-19 pandemic?
    a. Yes
    b. No
    c. NA

20. If yes, rate your concern for each of the following:
    a. Serious illness, impairment or death

b. Loss of health limiting my ability practice medicine
c. Loss of security in maintaining a job in medicine ("job security") and/or future promotion options
d. Transmitting COVID to my close family
e. Transmitting COVID to other friends and neighbors
f. Transmitting COVID to my medical co-workers, including colleagues, staff, trainees

21. Do you have enough personal protective equipment (PPE) to safely perform your job?
  a. Yes
  b. No
  c. NA
  d. No, what do you need access to (please specify):

22. On the whole, are you satisfied with your institution's response to COVID-19, independent of the state or city response?
  a. Yes
  b. No
  c. NA

23. If yes, what were the things that you believe your institution did very well in preparing your institution for the pandemic? (Please specify)

24. If no, in what ways are you not satisfied (Please specify)

25. Has your practice been changed by the COVID-19 pandemic?
  a. Yes
  b. No
  c. NA

26. Are you reducing the frequency of patient visits?
  a. Yes
  b. No
  c. NA
  d. If Yes, in what ways (please specify)

27. Are you refusing new patient visits as a result of COVID-19 pandemic?
  a. Yes
  b. No
  c. NA

28. Are you referring patients to other providers (local, or otherwise) to reduce their risk of COVID-19?
  a. Yes
  b. No
  c. NA

Society for NeuroOncology

29. Have you canceled what you would deem non-essential visits?
    a. Yes
    b. No
    c. NA

30. Have you canceled or pushed out patient visits?
    a. No
    b. Yes, by 2 weeks
    c. Yes, by up to 1 month
    d. Yes, by up to 2 months
    e. Yes, by more than 2 months
    f. If yes, what kind of patients (please specify):

31. Are these changes largely dictated by (check all that apply)
    a. Patients
    b. You
    c. Patient's Parents
    d. Your institution
    e. NA

32. Have you changed your MRI scheduling?
    a. Yes, up to 1 month delay
    b. Yes, up to 2 month delay
    c. Yes, more than 3 month delay
    d. No
    e. NA

33. Have you canceled or pushed out inpatient chemotherapy?
    a. Yes, up to 2 week delay
    b. Yes, up to 1 month delay
    c. Yes, beyond 1 month delay
    d. No
    e. NA

34. Have you used telephone or video visits?
    a. Yes
    b. No
    c. NA

35. In what percentage of patients in the last month have you used telephone visits?

36. In what percentage of patients in the last month have you used video visits?

Society for NeuroOncology

37. Was it difficult to transition to a virtual clinic?
    a. Yes, somewhat
    b. Yes, very difficult
    c. No
    d. NA
    e. If yes, why (please specify)

38. Did you get adequate IT support?
    a. Yes
    b. No
    c. NA

39. Have you been billing for telephone or video visits?
    a. Yes
    b. No
    c. NA

40. Is there significant pressure from your institution to keep in-person appointments mainly for billing purposes?
    a. Yes
    b. No
    c. NA

41. Have you received specific guidelines for effective use of and billing of telemedicine from your institution?
    a. Yes
    b. No
    c. NA

42. Have you ordered COVID-19 testing for your patients?
    a. Yes
    b. No
    c. NA

43. Are you being repurposed into a different role as a result of COVID-19?
    a. Yes
    b. No
    c. If yes, what role (please specify)

44. While it may be too early to tell, do you believe the survival outcomes due to practice changes in the treatment of your patients (not due to SARS-COV2 direct infection) will be deleteriously impacted because of the COVID-19 pandemic?
    a. Yes
    b. No

c. NA

45. Outside of the COVID-19 pandemic, do you typically enroll patients in clinical trials?
   a. Yes
   b. No

46. Have you suspended enrollment for any clinical trial?
   a. Yes
   b. No

47. Which clinical trial types have you suspended enrollment (check all that apply)?
   a. Phase I
   b. Phase II
   c. Phase I/II
   d. Phase III

48. What has been the primary reason have you suspended enrollment (check all that apply)?
   a. Risks outweigh potential benefit to patients
   b. Safety concerns of trial staff
   c. Safety concerns of clinical practitioners
   d. Investigational drug shortage
   e. Research coordinator availability
   f. Institution's mandate
   g. Other (please specify):

49. Do you believe primary brain tumor patients prior to any treatment are at increased risk for COVID-19?
   a. Yes
   b. No

50. If yes: check all tumor types are at increased risk:
   a. GBM
   b. anaplastic astrocytoma
   c. anaplastic oligodendroglioma
   d. grade II astrocytoma
   e. grade II oligodendroglioma
   f. pilocytic astrocytoma
   g. anaplastic meningioma
   h. atypical meningioma
   i. grade I meningioma
   j. Other (please specify):

51. Do you believe primary brain tumor patients off treatment are at increased risk for COVID-19?
   a. Yes

b. No

52. Do you believe typical steroid use in brain tumor patients increases susceptibility to COVID-19?
    a. Yes
    b. No
    c. Not sure, need more evidence

53. Do you believe temozolomide use increases susceptibility to COVID-19?
    a. Yes
    b. No
    c. Not sure, need more evidence

54. Do you believe immunotherapy use increases or decreases susceptibility to COVID-19?
    a. Yes, increases
    b. Yes, decreases
    c. No
    d. Not sure, need more evidence

55. Do you believe radiation increases susceptibility to COVID-19?
    a. Yes
    b. No
    c. Not sure, need more evidence

56. Is the ability to order MRIs reduced at your institution beyond your own ordering practice changes?
    a. Yes, somewhat
    b. Yes, largely
    c. No
    d. NA
    e. If yes, what has changed (please specify):

57. Are more MRIs being done outside of your institution during the pandemic?
    a. Yes, somewhat
    b. Yes, largely
    c. No
    d. NA

58. Are you worried about a reduction in quality
    a. Yes
    b. No
    c. NA

59. Have you noticed that you were discussing code status/end of life issues with your patients more frequently since the pandemic started? If yes, what was the change drive by?
    a. Yes, driven by my institution

Society for NeuroOncology

b. Yes, driven by my patients
c. Yes, driven by me
d. No
e. NA

60. Are you a:
    a. Neuro-Oncologist (from medical oncology, neurology or pediatric)
    b. Neurosurgeon
    c. Radiation Oncologist
    d. Advanced practice practitioner (nurse practitioner, physician assistant)
    e. Nurse
    f. Occupational, speech or physical therapist
    g. Scientist or researcher
    h. Trainee: medical student or resident
    i. Trainee: graduate student
    j. Trainee: clinical fellow
    k. Trainee: post-doctoral fellow
    l. Social worker
    m. Other (please specify):

61. Did Covid-19 result in elective surgical cases restriction in your practice?
    a. Yes
    b. No
    c. If yes, since when (please specify)

62. What percentage of your elective cases did you have to re-schedule for later?

63. What percentage of your elective cases did you have to cancel?

64. What percentage of your elective cases did you keep on schedule?

65. For those that you have cancelled, what were the typical reasons?
    a. Patient was COVID-19 positive
    b. Hospital/Institution did not allow elective surgeries
    c. Other (please specify)

66. Endoscopic endonasal cases:
    a. Not affected
    b. Only emergent cases allowed
    c. Institutional mandate to select alternative approaches, such as craniotomy

67. Cases potentially involving the mastoid air cells:
    a. Not affected
    b. Only emergent cases allowed

c. Institutional mandate to consider temporizing approaches, such as CSF shunting for hydrocephalus secondary to CP angle tumor

68. Awake craniotomies:
    a. Not affected
    b. Proceeding, no issues with use of LMA
    c. Proceeding, but without LMA
    d. NA

69. At your institution, the decision-making process regarding the triage of surgical neuro-oncology patients was guided by (check all that apply):
    a. National/International associations or authorities
    b. Division/Department choice
    c. Surgeon choice
    d. Other (please specify)

70. Did you have to perform surgical procedure/patient care without adequate PPE?
    a. Never
    b. At times
    c. Very often

71. Do you test your patients for Covid-19 prior to surgery for non-life-threatening emergent/urgent cases?
    a. Yes
    b. No

72. Do you have a dedicated operating room for Covid-19 positive patients?
    a. Yes
    b. No

73. During the Covid-19 outbreak, have you experienced storage in staff (check all that apply)?
    a. Neurosurgeon
    b. Residents
    c. Nurses
    d. Others
    e. No shortage experienced

74. Have you recommended BCNU wafers for your patients more frequently due to concerns for immunosuppression from systemic chemotherapy, during the COVID-19 pandemic?
    a. Yes
    b. No
    c. I don’t use BCNU wafers in my practice
    d. Other (please specify):

SNO
Society for NeuroOncology

75. Are you a neuro-oncology fellowship program director?
    a. Yes
    b. No

76. Are you altering standard of care?
    a. Yes
    b. No

77. Are temozolomide regimens being altered?
    a. Yes
    b. No
    c. If yes, (please specify)

78. Are you obtaining lab work more frequently for monitoring of immune status?
    a. Yes
    b. No

79. Are you changing steroid regimens because of immunosuppression concerns?
    a. Yes
    b. No
    c. If yes, (please specify)

80. Do you believe this is having a clinical impact?
    a. Yes
    b. No
    c. If yes, (please specify)

81. Are you more careful using more myelotoxic regimens (CCNU, carboplatin)
    a. Yes
    b. No
    c. If yes, (please specify)

82. What about infusions like bevacizumab? Have you changed the dosing/frequency of administration?
    a. Yes
    b. No
    c. If yes, (please specify)

83. Are observation MRIs being pushed out for GBM patients, if so, how far out for now?
    a. Yes
    b. No
    c. If yes, what was the change in frequency (please specify)

84. What is your approach to tumor treating field therapy (TTF) for GBM patients during pandemic?
    a. Stop (for active patients)

b. Continue as before (for active patients)
c. Not offer to new patients
d. I don't prescribe TTF

85. How do you manage patients requiring in-patient chemotherapy?
  a. No change in plan
  b. Deferred treatment
  c. Change to other treatment

86. Did you have to cancel/postpone HD chemotherapy with ASCT for PCNSL patients at your institution?
  a. Yes
  b. No
  c. If yes, how many patients (please specify)

87. Did you alter your practice for patients requiring intrathecal chemotherapy?
  a. Yes
  b. No
  c. If yes, (please specify)

88. Have you seen any COVID-19 positive patients in your practice? Are you aware of any of your patients testing positive for COVID-19?
  a. Yes
  b. No
  c. If yes, how many (please specify)

89. Have there been changes to how your run your clinical trials and how patients on clinical trials are being treated?
  a. Yes
  b. No

90. Did you receive instructions from consortia and/or sponsors and your IRB regarding clinical trial conduct during the pandemic? (How to deal with deviations, etc.).
  a. Yes
  b. No

91. Are you significantly reducing or stopping regimens that are off-label/not proven?
  a. Not applicable – I seldom use
  b. Yes, somewhat reducing frequency
  c. Yes, mainly stopping
  d. No

92. Have you noticed increased need for palliative care consultations in your patients during the pandemic?

Society for NeuroOncology

a. Yes
b. No

93. Have you noticed increased level of anxiety and depression in your patient population during the pandemic?
    a. Yes
    b. No

94. If you are a pediatric neuro-oncologist, have you faced any specific challenges related to COVID-19 in your practice?
    a. Yes
    b. No
    c. NA
    d. If yes, (please specify)

95. If you are a pediatric neuro-oncologist, did you have to significantly alter your treatment plans?
    a. Yes
    b. No
    c. NA
    d. If yes, (please specify)

96. Have you recommended BCNU wafers for your patients more frequently due to concerns for immunosuppression from systemic chemotherapy, during the COVID-19 pandemic?
    a. Yes
    b. No
    c. I don't use BCNU wafers in my practice
    d. Other (please specify)

97. Are you a neuro-oncology fellowship program director?
    a. Yes
    b. No

98. Have you modified treatment regimens for glioma patients as a response to COVID-19?
    a. Yes
    b. No

99. If yes, click all that apply to your modified regimens:
    a. Shorter course, slightly higher dose
    b. Shorter course, lower dose
    c. Other (please specify)

100. In what percentage of your cases have you modified the treatment plan?

Society for NeuroOncology

101. At your institution, the decision-making process regarding the modification of radiotherapy for your patients was guided by (check all that apply):
    a. National / International associations or authorities
    b. Local hospital governance/policy
    c. Division/Department choice
    d. Physician's choice
    e. Published literature
    f. Other (please specify)

102. Have you delayed start times for involved field radiation for your high-grade glioma patients?
    a. Yes, on average by less than 2 weeks
    b. Yes, on average by 2-4 weeks
    c. Yes, on average by more than 4 weeks
    d. No, for the most part.

103. Have you delayed start times for involved field radiation for other indications?
    a. Yes, on average by less than 2 weeks
    b. Yes, on average by 2-4 weeks
    c. Yes, on average by more than 4 weeks
    d. No, for the most part.

104. Have you modified stereotactic radiosurgery plans for your CNS metastatic disease patients?
    a. Yes
    b. No
    c. Yes, (please specify how)

105. Has your lab been closed?
    a. Yes
    b. No
    c. NA

106. Is it closed for non-essential experiments?
    a. Yes
    b. No
    c. NA

107. Do you feel pressured beyond your own desires to be in the lab?
    a. Yes
    b. No
    c. NA

108. Are you fearful for you and/or your family's health by your research duties due to the COVID-19 pandemic?
    a. Yes

b. No
c. NA

109. Are you satisfied with your institution’s response to the pandemic?
a. Yes
b. No
c. NA
d. No, what should be improved (please specify)

110. Did you have to stop ongoing long-term experiments (e.g. sacrifice mice, terminate cell cultures, etc.)?
a. Yes
b. No
c. NA

111. Are you having difficulty receiving patient tumor tissue for ongoing/continuing projects?
a. Yes
b. No
c. NA

112. Do you think your research will advance further than it would have in any way by taking time away from the lab? (Check all that apply)
a. Yes, it will give you time to read relevant articles
b. Yes, it will give you time to study computational tools
c. Yes, it will give you time to write grant proposals
d. Yes, it will give you time to write manuscripts
e. Yes, it will give you time to learn/get acquainted with new techniques
f. No
g. NA

113. Has any of your funding been suspended?
a. Yes
b. No
c. NA
d. Yes, (please specify the source of funding; government, private sector, foundations)

114. Are any of your collaborators furloughed or had to be fired?
a. Yes
b. No
c. NA

115. Is your research at risk of failing?
a. Yes
b. No

Society for NeuroOncology

c. I don't know/too early to tell
d. NA

116. Have your grant submission deadlines been postponed?
    a. Yes
    b. No
    c. Yes (please specify the agencies that postponed the deadlines; government, private sector, foundations)

117. Are you worried about your future research funding given the strain the pandemic has on resources and the economy in general?
    a. Yes, mildly concerned
    b. Yes, moderately concerned
    c. Yes, very concerned
    d. No
    e. NA

118. Will this pandemic alter your academic career?
    a. Yes
    b. No
    c. Too early to tell
    d. NA

119. Are you at risk of losing your Visa status due to the pandemic?
    a. Yes
    b. No
    c. Too early to tell
    d. NA

120. What percentage of your educational seminars and meetings have been shifted to video learning?

121. What percentage of your educational seminars and meetings have been canceled?

122. How did your responsibilities/rotations change during the pandemic?
    a. No change
    b. Change (please specify)

123. Do you think your clinical training will advance any further than it would have in any way during the COVID-19 pandemic?
    a. Yes
    b. No
    c. NA

Society for NeuroOncology

124. Has your experience with neuro-oncology patients during COVID-19 impacted your interest in pursuing a career in neuro-oncology?
    a. Yes – less interested in neuro-oncology
    b. Yes – more interested in neuro-oncology
    c. No
    d. NA

125. Do you think you will be less prepared as a neuro-oncologist due to time lost during COVID-19 pandemic?
    a. Yes
    b. No
    c. NA

126. Was your recruitment for 2021 positions affected by the pandemic?
    a. Yes
    b. No

127. Did you interview all interested candidates in time for the Match list submission?
    a. Yes
    b. No

128. Did you use virtual tools (Skype, Zoom, etc.). for the interview process?
    a. No
    b. Yes (what percentage)?

129. Are you worried you may not fill your fellowship slots because of the pandemic?
    a. Yes
    b. No

130. Are you worried you may lose funding to support Neuro-Oncology fellows in the future as a result of budget cuts secondary to the pandemic?
    a. Yes
    b. No

131. Did you receive more consultations for Neuro-Oncology patients during COVID-19 pandemic?
    a. Yes
    b. No
    c. NA
    d. If yes, what were the main reasons for consultations? (please specify)

132. Did you have to suspend support group activities during the pandemic?
    a. Yes
    b. No
    c. NA

Society for NeuroOncology

133. If you had to suspend in-person support group activities, have you converted your support group into a virtual platform?

a. Yes
b. No
c. Other (please specify)

134. What other challenges have you faced during the COVID-19 pandemic?
